# Retracted: Detection of Abnormal Hemoglobin Variants by HPLC Method: Common Problems with Suggested Solutions

**DOI:** 10.1155/2015/498230

**Published:** 2015-04-16

**Authors:** International Scholarly Research Notices

The paper titled “Detection of Abnormal Hemoglobin Variants by HPLC Method: Common Problems with Suggested Solutions,” [1] published in International Scholarly Research Notices, has been retracted as it was submitted for publication without the knowledge and approval of one of the coauthors, Dr. Suman Lata Mendiratta. The submitting author Dr. Dipti Kalita plagiarized data from the Thalassemia Control Project headed by Dr. Suman Lata Mendiratta.
